# Distinction of early complement classical and lectin pathway activation *via* quantification of C1s/C1-INH and MASP-1/C1-INH complexes using novel ELISAs

**DOI:** 10.3389/fimmu.2022.1039765

**Published:** 2022-11-04

**Authors:** Lisa Hurler, Erik J. M. Toonen, Erika Kajdácsi, Bregje van Bree, Ricardo J. M. G. E. Brandwijk, Wieke de Bruin, Paul A. Lyons, Laura Bergamaschi, Cambridge Institute of Therapeutic Immunology and Infectious Disease-National Institute of Health Research (CITIID-NIHR) COVID BioResource Collaboration, Stephen Baker, György Sinkovits, László Cervenak, Reinhard Würzner, Zoltán Prohászka

**Affiliations:** ^1^ Department of Internal Medicine and Haematology, Semmelweis University, Budapest, Hungary; ^2^ Research and Development Department, Hycult Biotech, Uden, Netherlands; ^3^ Cambridge Institute of Therapeutic Immunology and Infectious Disease, Jeffrey Cheah Biomedical Centre, University of Cambridge, Cambridge, United Kingdom; ^4^ Department of Medicine, University of Cambridge, Addenbrooke’s Hospital, Cambridge, United Kingdom; ^5^ Institute of Hygiene and Medical Microbiology, Medical University of Innsbruck, Innsbruck, Austria; ^6^ Research Group for Immunology and Haematology, Semmelweis University – Eötvös Loránd Research Network (Office for Supported Research Groups), Budapest, Hungary

**Keywords:** early complement activation, classical pathway activation, lectin pathway activation, C1-INH complexes, assay development and validation, C1s/C1-INH complex, MASP-1/C1-INH complex

## Abstract

The most commonly used markers to assess complement activation are split products that are produced through activation of all three pathways and are located downstream of C3. In contrast, C4d derives from the cleavage of C4 and indicates either classical (CP) or lectin pathway (LP) activation. Although C4d is perfectly able to distinguish between CP/LP and alternative pathway (AP) activation, no well-established markers are available to differentiate between early CP and LP activation. Active enzymes of both pathways (C1s/C1r for the CP, MASP-1/MASP-2 for the LP) are regulated by C1 esterase inhibitor (C1-INH) through the formation of covalent complexes. Aim of this study was to develop validated immunoassays detecting C1s/C1-INH and MASP-1/C1-INH complex levels. Measurement of the complexes reveals information about the involvement of the respective pathways in complement-mediated diseases. Two sandwich ELISAs detecting C1s/C1-INH and MASP-1/C1-INH complex were developed and tested thoroughly, and it was investigated whether C1s/C1-INH and MASP-1/C1-INH complexes could serve as markers for either early CP or LP activation. In addition, a reference range for these complexes in healthy adults was defined, and the assays were clinically validated utilizing samples of 414 COVID-19 patients and 96 healthy controls. The immunoassays can reliably measure C1s/C1-INH and MASP-1/C1-INH complex concentrations in EDTA plasma from healthy and diseased individuals. Both complex levels are increased in serum when activated with zymosan, making them suitable markers for early classical and early lectin pathway activation. Furthermore, measurements of C1-INH complexes in 96 healthy adults showed normally distributed C1s/C1-INH complex levels with a physiological concentration of 1846 ± 1060 ng/mL (mean ± 2SD) and right-skewed distribution of MASP-1/C1-INH complex levels with a median concentration of 36.9 (13.18 - 87.89) ng/mL (2.5-97.5 percentile range), while levels of both complexes were increased in COVID-19 patients (p<0.0001). The newly developed assays measure C1-INH complex levels in an accurate way. C1s/C1-INH and MASP-1/C1-INH complexes are suitable markers to assess early classical and lectin pathway activation. An initial reference range was set and first studies showed that these markers have added value for investigating and unraveling complement activation in human disease.

## Introduction

**Figure 1:**
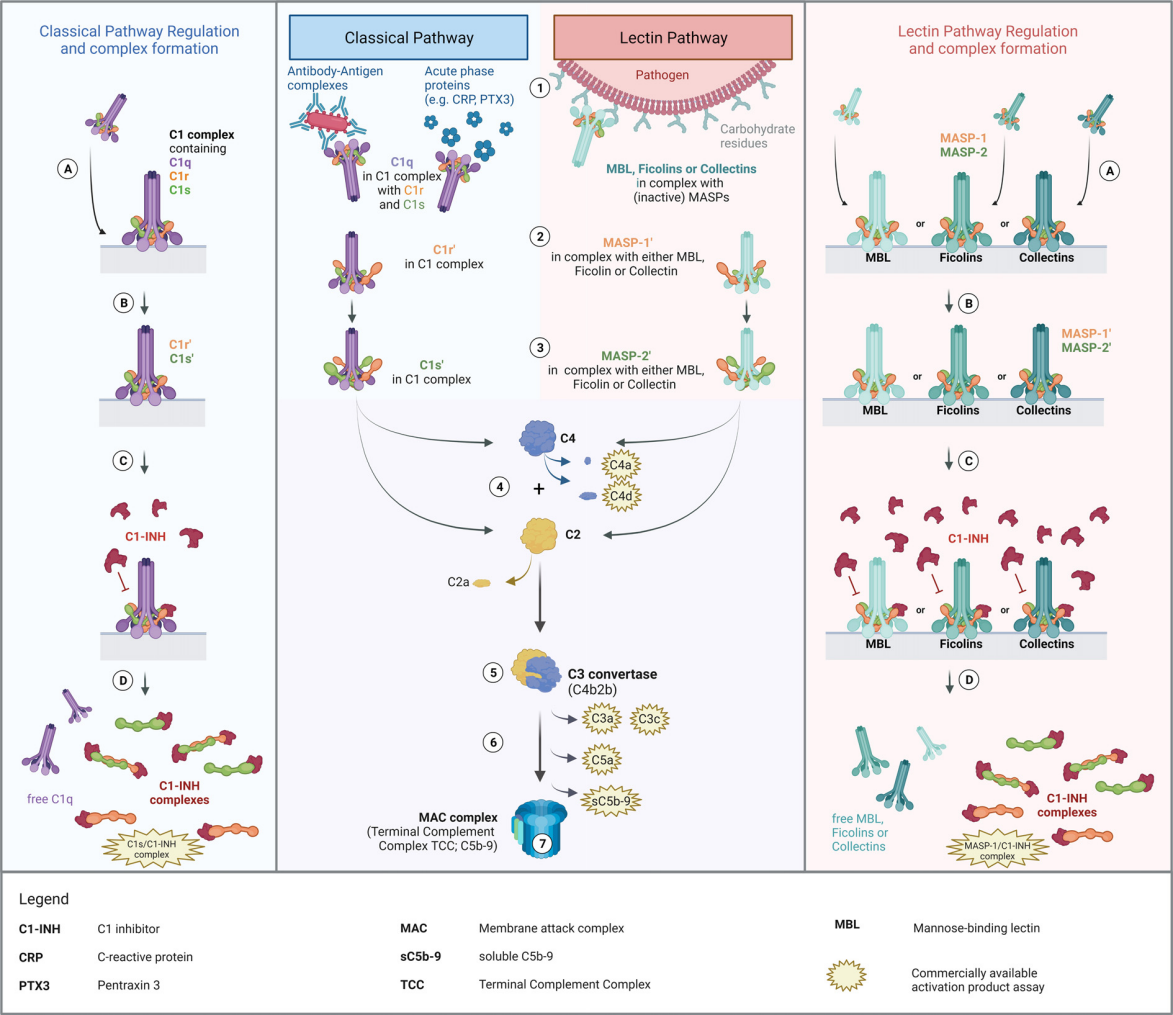
Overview of Classical and Lectin Pathway activation, regulation by C1-INH and proposed mechanism of complex formation. Middle panel: Activation of the classical and the lectin pathway (1). Recognition of triggering compound by the respective recognition molecules (C1q for classical pathway; MBL, ficolins or collectins for lectin pathway) and binding thereof to the activating compounds/structures. (2) Zymogen autoactivation of the first serine protease after structural changes of the associated recognition molecules upon binding to activating structures. (3) Catalytically activation of the second serine proteases by the activated first serine proteases. (4) Cleavage of C2 and C4 by activated serine proteases. (5) Formation of the classical C3 convertase, consisting of C4b and C2b [updated nomenclature according to (3)]. (6) Downstream complement activation going along with the release of several complement activation/split products. (7) Formation of the Terminal Complement Complex (TCC) or the Membrane Attack Complex (MAC complex), when formed on a membrane, and lysis or cell clearance. Left and right panel: Regulation of classical and lectin pathway activation by C1-INH and proposed theory of complex formation. (A) Binding of the pattern recognition molecule to an activating surface, resulting in conformational changes in the respective protein complexes. (B) Activation of serine proteases, allowing downstream complement activation. (C) Regulation of pathway activation by C1-INH through covalent binding to active sites of the serine proteases, blocking protease function and further activation of the respective pathways. (D) Dissociation of the complexes upon binding of C1-INH and release of C1-INH complexes as well as free pattern recognition molecules into the circulation. The figure was created with BioRender.com.

The complement system is a proteolysis-based activation cascade, consisting of more than 40 plasma proteins, which acts as a first line defense in the fight against microorganisms such as bacteria, viruses or fungi. Besides, the complement system also plays a role in the clearance of damaged or altered host cells (1), in enhancing the adaptive immune response (2), and in autoimmune diseases. It can be activated *via* three different pathways, the classical (CP), the lectin (LP), and the alternative pathway (AP). An overview of classical and lectin pathway activation as well as a proposed scheme of complex formation is demonstrated in **Figure 1**.

The classical pathway is activated by binding of the recognition protein C1q, part of the C1 complex (C1qC1r_2_C1s_2_), to circulating antibody-antigen complexes containing IgG or IgM. Activation can also occur in an immune complex-independent manner, through binding of C1q to acute phase proteins like the C-reactive protein or Pentraxin 3 (4, 5). Additionally, there is also the possibility of direct interaction between the initiator C1q and viral proteins, including surface proteins of HIV-1 and EBV (6), lipopolysaccharides such as on the surface of bacteria (7), and apoptotic cells (8). Upon binding of the C1 complex to an activating target surface, structural changes occur in the C1q, which trigger the associated zymogen C1r in the C1 complex to autoactivate. Auto-activated C1r can subsequently activate proenzyme C1s (9), which then allows proteolytic cleavage of C2 and C4, initiating the formation of the classical C3 convertase (C4b2b) and downstream complement activation. The lectin pathway is activated in a slightly different way. While classical pathway activation can only be initiated by C1q, the lectin pathway can be activated through several pattern recognition molecules: collectins, such as the mannan-binding lectin (MBL), collectin-10, and collectin-11; and the three ficolins, ficolin-1, ficolin-2 and ficolin-3 (10). Upon recognition of triggering carbohydrate structures or acetyl group patterns on pathogen surfaces, both the MBL-associated serine protease 1 (MASP-1) and -2 (MASP-2) are activated. While activated MASP-2 can cleave C4 and C2, MASP-1 can only cleave C2 as a central component of the complement cascade, and not C4 (11). However, MASP-1 can also cleave MASP-2, thereby additionally enhancing C4 and C2 cleavage (12, 13). Activation of the CP and LP and subsequent cleavage of C2 and C4 will lead to the formation of C3 and C5 convertases and downstream complement activation. Finally, this results in the formation of the terminal complement complex (TCC), also known as the membrane attack complex (MAC complex), when formed on the membrane of a target cell (14). After complement activation, the targeted cell will either be osmotically lysed or opsonized and eliminated by phagocytosis (15).

For assessing complement activation, several well-known and widely used biomarkers are available (see **Figure 1**). Most commonly used markers for measuring complement activation in human plasma are C3 split products (anaphylatoxin C3a, C3c and C3d), C4 split products C4a and C4d, the alternative pathway activation product Bb (not shown in **Figure 1**), the anaphylatoxin C5a and the terminal pathway activation product sC5b-9 (16). C3 split products, such as the anaphylatoxin C3a, C3c and C3d, as well as the terminal complement complex (TCC/sC5b-9) are produced as a result of activation of all three complement pathways. As an example, C3d is a robust marker for C3 activation, extensively studied in complement-mediated diseases such as age-related macular degeneration (AMD) or Systemic lupus erythematosus (SLE) (17, 18). In contrast, C4d is derived from the cleavage of C4 and hence indicates both classical and lectin pathway but not alternative pathway activation. Besides that, many C3d and C4d assays are widely used in routine diagnostics and available commercially, especially investigation of C4d is mainly used for immunostaining in (kidney) biopsies (19–21). Although C4d is perfectly able to distinguish between CP/LP and AP activation, no well-established markers to measure early classical and early lectin pathway activation are available. Information thereof could provide further insights into - and better understanding of - the role of complement in health and disease. Next to that, it might have added value for the diagnosis of complement-mediated disease as well as for monitoring disease onset and severity. Additionally, knowledge about the early CP and LP activation state could help in the development and monitoring of complement inhibitors specifically targeting early components like C1s (22, 23).

Activation of complement *via* both the CP and LP is tightly regulated by C1 esterase inhibitor (C1-INH), a highly glycosylated protein encoded by the *SERPING1* gene, predominantly expressed in the liver (24). C1-INH is the only known regulator of the early classical and lectin pathways, while it also has a profound role in regulating the contact system, the fibrinolytic system as well as the coagulation system (25). With regard to the CP, it can covalently bind to activated C1r and C1s, blocking the serine protease function and hence inhibiting proteolytic cleavage of C4 and C2 and further CP activation (26). Upon binding of C1-INH, the C1 complex dissociates and releases free C1q as well as covalent C1r/C1-INH and C1s/C1-INH complexes (27). Those complexes are internalized and degraded by the low density lipoprotein receptor-related protein rapidly after release into the circulation (28). C1-INH also regulates the lectin pathway as it forms covalent complexes by directly binding activated MASP-1 and MASP-2, thereby downregulating pathway activation.

For both protein complexes, several studies have reported that levels in circulation are associated with human disease. Regarding C1s/C1-INH, levels were increased 2-3-fold in Hereditary angioedema (HAE) patients when compared to controls (29, 30). In addition, increased C1s complex levels were also reported for other diseases involving classical pathway activation, such as Systemic lupus erythematosus (SLE), glomerulonephritis, and rheumatoid arthritis (RA) (31, 32). Concerning MASP/C1-INH complexes, a given amount of both MASP-1/C1-INH and MASP-2/C1-INH naturally exists in the circulation (33). However, lower MASP-1/C1-INH complex levels were reported in HAE patients with decreased C1-INH activity (33), whereas type II HAE patients seem to have increased MASP-1/C1-INH levels when compared to healthy controls (30). These results suggest that C1s/C1-INH and MASP/C1-INH protein complexes are involved in the pathogenesis of several complement-mediated diseases and that they may have added value as biomarkers for diagnosis and disease- or treatment monitoring. While the specific function of the C1-INH complexes is not known yet and requires further research, complex formation of CP and LP serine proteases with C1-INH does require activation of the respective serine proteases (like C1s, C1r, MASP-1 or MASP-2), making the complexes promising biomarkers to distinguish between early classical and early lectin pathway activation. Higher levels of the C1-INH complexes in disease might indicate higher levels of complement regulation through C1-INH and hence also higher activation levels of the respective pathways.

So far, no commercially available assays exist in order to measure C1s/C1-INH complex and MASP-1/C1-INH complex concentrations in a reliable and standardized way, making it difficult to compare already published studies about C1-INH complexes. In this study, we aimed to develop two sensitive and specific immunoassays for quantifying C1s/C1-INH and MASP-1/C1-INH complexes in human blood samples (plasma and serum). In addition, we intended to show that C1-INH complex levels increase upon *in-vitro* activation of the respective pathways in human serum, making them suitable biomarkers for indirect measurement of ongoing activation of either the classical pathway (C1s/C1-INH complex) or the lectin pathway (MASP-1/C1-INH complex).

A further aim was to clinically validate these complexes also *in vivo*. Therefore, we investigated C1-INH complex levels in COVID-19 patients, where complement activation is known to play a role in the pathomechanism of the disease, and compared the results to C1-INH complex concentrations measured in healthy controls’ samples.

## Material and methods

### Patient cohort

A total of 414 COVID-19 patients (34, 35) and 96 healthy controls (34, 36) were enrolled and sampled as described elsewhere. In brief, whole blood was collected into EDTA-treated tubes, before cells and plasma were separated by centrifugation. EDTA-plasma samples were stored in aliquots at -80°C until further usage.

Ethical approval was obtained from the East of England – Cambridge Central Research Ethics Committee (“NIHR BioResource” REC ref 17/EE/0025, and “Genetic variation AND Altered Leucocyte Function in health and disease – GANDALF” REC ref 08/H0308/176), the Hungarian Ethical Review Agency (ETT-TUKEB; No. 8361-1/2011-EKU and IV/4403-2/2020/EKU) and the Government Office of the Capital City Budapest (31110-7/2014/EKU (481/2014)), based on the position of the Medical Research Council. The studies were conducted in accordance with the Declaration of Helsinki. Written informed consent to participate in this study was provided by the participants or their closest relative available.

### Biological samples

Matching sample sets, consisting of citrate plasma, heparin plasma, EDTA plasma and serum each, of ten healthy individuals were purchased from BioIVT (BioIVT, New York, USA), stored in aliquots at -80°C and included in measurements during the assay development. C1s/C1-INH complex and C1s enzyme were purchased from CompTech (Complement Technology Inc., Texas, USA), while C1q was purchased from Quidel (Quidel Corporation, San Diego, USA).

Recombinant serine proteases (C1r, C1s, MASP-1, MASP-2) and C1-INH were prepared and purified in-house as described elsewhere (30). Blood samples from animals were obtained from commercial sources. Murine, pig and dog sera were ordered from Innovative (Innovative Research Inc., Michigan, USA), while rat and horse sera were purchased from Harlan (Harlan Bioproducts for Science Inc., Maryland, USA). For complement activation experiments, complement-preserved normal human serum (NHS) was prepared freshly from a pool of 12 healthy individuals and stored in aliquots at -80°C until further usage.

### Production of monoclonal antibodies against C1s, MASP-1 and C1-INH

C1-INH was further purified from human plasma C1-INH concentrate by ion-exchange chromatography and size exclusion chromatography (30). Recombinant CCP1-CCP2-SP fragments of C1s and MASP-1 were produced and purified as previously published (37, 38).

Eight-to-ten-week-old BALB/c mice were repeatedly immunized (100 μg antigen/mouse in complete Freund’s adjuvant subcutaneously, 50 μg antigen/mouse in incomplete Freund’s adjuvant subcutaneously, finally, 50 μg antigen/mouse in PBS intravenously). Mice with high serum antibody titer against C1s, MASP-1 or C1-INH were sacrificed, the spleens were removed and splenocytes were fused with Sp2/0-Ag14 myeloma cells in the presence of PEG solution (Hybri-Max, Sigma-Aldrich, Budapest, Hungary). Selection was performed by standard HAT supplemented DMEM (Sigma-Aldrich, Budapest, Hungary) (39). Clones were tested for specificity and cross-reactivity by direct antigen ELISA, and for isotype by sandwich ELISA (data not shown).

### *In silico* analysis of amino acid sequence

Similarity of amino acid sequences of the complex components (C1s, MASP-1 and C1-INH) and other human proteins was tested *in silico* using *Homo sapiens* protein BLAST (https://blast.ncbi.nlm.nih.gov). More than 60% agreement in the amino acid sequence between the tested protein and any other protein sequence in the database was chosen as an arbitrary cut off for high similarity and did require experimental specificity testing of the developed antibodies in a direct ELISA setup.

### Direct ELISA to test specificity of antibodies

The specificity of in-house produced antibodies for complement proteins was tested by direct ELISA. Relevant proteins of the respective pathways (either recombinant or commercial ones purified from human serum, dependent on availability) were immobilized at a concentration of 1 μg/mL in bicarbonate buffer (0.1 M bicarbonate, pH 9.6; 100 μL/well) on Nunc Maxisorp 96 well plates (Thermo Fisher Scientific, Waltham, USA) at 4°C over night. Wells were blocked with 2% BSA in PBS for 1.5 h at room temperature. After blocking, plates were washed with PBS-Tween. Primary monoclonal antibodies (anti C1-INH, anti C1s or anti MASP-1 used in the complex assays) were added at a concentration of 3 μg/mL in dilution buffer from the complex assays and incubated for 1h at room temperature. After washes (4x), HRP-conjugated goat anti-mouse antibody (Southern Biotech, Birmingham, USA) was added (1:4000 in dilution buffer of the complex assays) and incubated for 1 h at room temperature. Following washes, 50% TMB in H_2_O was added, the plate was incubated for 5 min before the reaction was stopped using Oxalic acid and the absorbance was measured at 450 nm on a plate reader. Measurements were done in duplicate, while the experiment was performed in triplicate.

### Preparation of in-house MASP-1/C1-INH complexes

MASP-1/C1-INH complexes were prepared in-house as described before (30). In short, recombinantly produced activated MASP-1 (CCP1-CCP2-SP domain) was incubated in a 1:1 molar ratio with ultra-pure C1-INH (purified by anion exchange chromatography) for 2 h at 37°C in PBS. After incubation, complex formation was validated using SDS page and complexes were stored in 1% BSA-PBS at -80°C until further usage, while repeated freeze-thawing was prevented through aliquoting.

### Development of C1s/C1-INH and MASP-1/C1-INH complex assays and assay performance

Two novel immunoassay detecting levels of C1s/C1-INH (cat #HK399) and MASP-1/C1-INH (cat# HK3001) complex *in vitro* were developed. Plasma samples (citrate, heparin and EDTA), serum samples and standards were incubated in wells coated with monoclonal antibodies recognizing either the serine proteases C1s or MASP-1 of the complex. After incubation and washing, wells were incubated with an HRP-labeled monoclonal antibody detecting bound C1-INH in the complexes. Addition of tetramethylbenzidine (TMB) substrate started an enzymatic reaction thereby producing a colored product. The reaction was stopped by adding oxalic acid and the absorbance at 450 nm (OD450 nm) was measured using a spectrophotometer. This protocol was used to evaluate several assay characteristics such as (but not limited to) sensitivity, specificity, parallelism between calibrator and samples, matrix effects, recovery, intra- and inter variability and stability of samples and calibrators. All assay development aspects were evaluated using the following general requirements:

- Max. optical density at 450 nm (OD450 nm) ≤ 3.0,
- OD450 nm highest concentration standard/calibrator (S1) 1.7<OD450 nm< 3.0,
- OD450 nm blank ≤ 0.2, signal to noise ratio (S/N) > 10.

Variation between samples or conditions was evaluated by calculating the coefficient of variation.


$$\%CV=\;\frac{standard\;deviation\;\left(SD\right)}{mean}x100$$

### Recovery of C1-INH complexes in EDTA plasma

To evaluate recovery of C1-INH complexes in EDTA plasma, samples of three individuals with different, but previously determined, complex concentrations (low, middle, and high) were mixed in different percentages/ratios (100–0, 75–25, 50–50, 25–75, 0–100) and incubated for 30 min at room temperature. Next, C1s/C1-INH complex and MASP-1/C1-INH complex concentrations were measured in these mixed samples. Recovery was determined according to following equation in which the expected concentration was compared to the observed (measured) concentration:


$$recovery\;\left[\%\right]=\frac{measured\;concentration\;of\;mixed\;sample}{expected\;concentration\;\left[\left(percentage\;x\;value\;sample\;A\right)+\left(percentage\;x\;value\;sample\;B\right)\right]}x100$$

In general, requirements were met if recovery was between 80% - 120%.

### Specificity of the assays

The specificity or cross-reactivity of the C1-INH complex assays was tested in two ways. Firstly, cross-reactivity with other proteins or family members and the uncomplexed complement components was investigated. Uncomplexed complement components (C1s and C1-INH for the C1s/C1-INH complex, and MASP-1 and C1-INH for the MASP-1/C1-INH complex) were diluted in dilution buffer and signals were measured in the C1-INH complex assays as described above. Secondly, it was also investigated whether the assays show cross reactivity with species other than human. Serum samples of animal origin (mouse, rat, pig, horse and dog) were measured 10x less diluted compared to dilutions used for human samples.

### Inter- and intra-assay variation

Intra-assay variation (multiple determinations of single samples within a single test run) was tested by the measurement of three independent aliquots each of four different samples within one test run. The experiment was conducted separately by two different operators and means and coefficients of variation (%CV) were calculated from the aliquots tested. Inter-assay variation (multiple determinations of single samples in several assay runs) was determined by calculation of the means and %CVs between the test runs from both operators.

A coefficient of variation <10% indicates low variation for intra-assay variation, while a coefficient of variation <20% indicates low variation for inter-assay variation.

### Stability testing of C1-INH complexes

During development, both benchtop and freeze-thaw stability in samples were evaluated. Benchtop stability of the C1-INH complexes was assessed by incubating undiluted samples for different time intervals at room temperature as well as on ice, before those samples were measured in the assays as described above. The measured complex concentration of a sample kept at the respective condition for 10 minutes served as a control. Freeze-thaw stability was evaluated by repeatedly freezing (-80°C) and thawing individual aliquots of the samples for up to 4 cycles and comparing the measured C1-INH complex concentrations to concentrations determined in an unthawed aliquot. When performing the freeze-thawing steps, samples were thawed at room temperature, kept on ice for 10 min and subsequently frozen at -80°C. Stability testing of the complexes was done in EDTA plasma, citrate plasma as well as in purified form (commercially available C1s/C1-INH complex or in-house MASP-1/C1-INH complex). Changes of the complex concentration between 80-120% compared to the controls (10 min sample for the benchtop stability, unthawed sample for the freeze-thaw stability) were considered as acceptable.

### Preparation of zymosan activated serum

Zymosan was boiled in PBS for 1 h, and subsequently washed 4 times in PBS. After washing, zymosan was mixed to a final concentration of 10 mg/mL with 2% complement-preserved normal human serum (NHS) in VBS^++^ buffer (veronal-buffered saline containing 0.15 mM Ca^2+^ and 1 mM Mg^2+^), before tubes were incubated at 37°C on a shaker (250 RPM). Samples were taken directly after mixing and after incubation for 5 min, 10 min, 20 min, 30 min, 1 h, 2 h, 3 h and 5 h. Directly after sampling, each sample was supplemented with EDTA to a final concentration of 10 mM, in order to avoid further artificial complement activation. Remaining zymosan was removed by centrifugation and the supernatant was stored at -80°C until further usage. When measuring C1-INH complex concentrations, samples were diluted further (3x for the MASP-1/C1-INH complex assay and 30x for the C1s/C1-INH assay) to measure in the reliable range of the assays, and levels of C1s/C1-INH and MASP-1/C1-INH complex were determined as described above.

Several controls were included: Auto-activation of the classical and lectin pathway was measured in NHS without addition of either EDTA or zymosan, one control sample contained NHS and 10 mM EDTA as a negative control (NHS+EDTA) and another sample contained all three components (NHS+zymosan+EDTA).

### Specific activation of CP or LP

For specific activation of either the classical or the lectin pathway, complement-preserved normal human serum (NHS) was activated in a final concentration of 2% on WIESLAB^®^ Complement System Screen plates (SVAR Life Science; WIESLAB^®^ Complement System Classical Pathway (COMPLCP310RUO) for specific CP activation, WIESLAB^®^ Complement System MBL Pathway (COMPLMP320RUO) for specific LP activation). Samples were activated for 5 min, 10 min, 20 min, 30 min, 1 h, 2 h and 3 h. After activation, the samples were collected from the wells, stored at -80°C, and used for C1s/C1-INH and MASP-1/C1-INH complex measurements at a later time point. Both complexes were determined as described before.

Subsequently, the level of complement pathway activity (as measured by C9 neoepitope formation during activation) was determined according to the manufacturer’s instructions. Results were expressed as percentages calculated by the OD values measured in positive and negative controls, as suggested by the SVAR kits protocol.

### Statistical analysis

GraphPad Prism 9 was used for statistical analysis and for data visualization. Comparisons of C1-INH complex levels in two different groups were performed either using the Mann-Whitney U or the one sample t-test when appropriate. For the zymosan and CP/LP activation, influence of incubation time and added reagents (zymosan, EDTA or both) or coating (IgM or mannan) on C1-INH complex concentrations was tested by performing a two-way ANOVA. If not stated otherwise, concentrations are given as mean ± SD. Normal distribution of complex levels was tested using the D’Agostino& Pearson test. For normally distributed values, the reference range was calculated using mean ±2SD. For skewed distribution of values, the reference range was reported using median (2.5 percentile range - 97.5 percentile range).

In all statistical tests a p-value<0.05 was considered statistically significant (* p<0.05, ** p<0.01, *** p<0.001, **** p<0.0001).

## Results

### Development and characterization of sandwich ELISAs measuring C1s/C1-INH complex and MASP-1/C1-INH complex

Two new immunoassays have been developed, measuring either C1s/C1-INH or MASP-1/C1-INH complex levels, both formed during complement regulation by binding of C1-INH to activated serine proteases.

**Figure 2:**
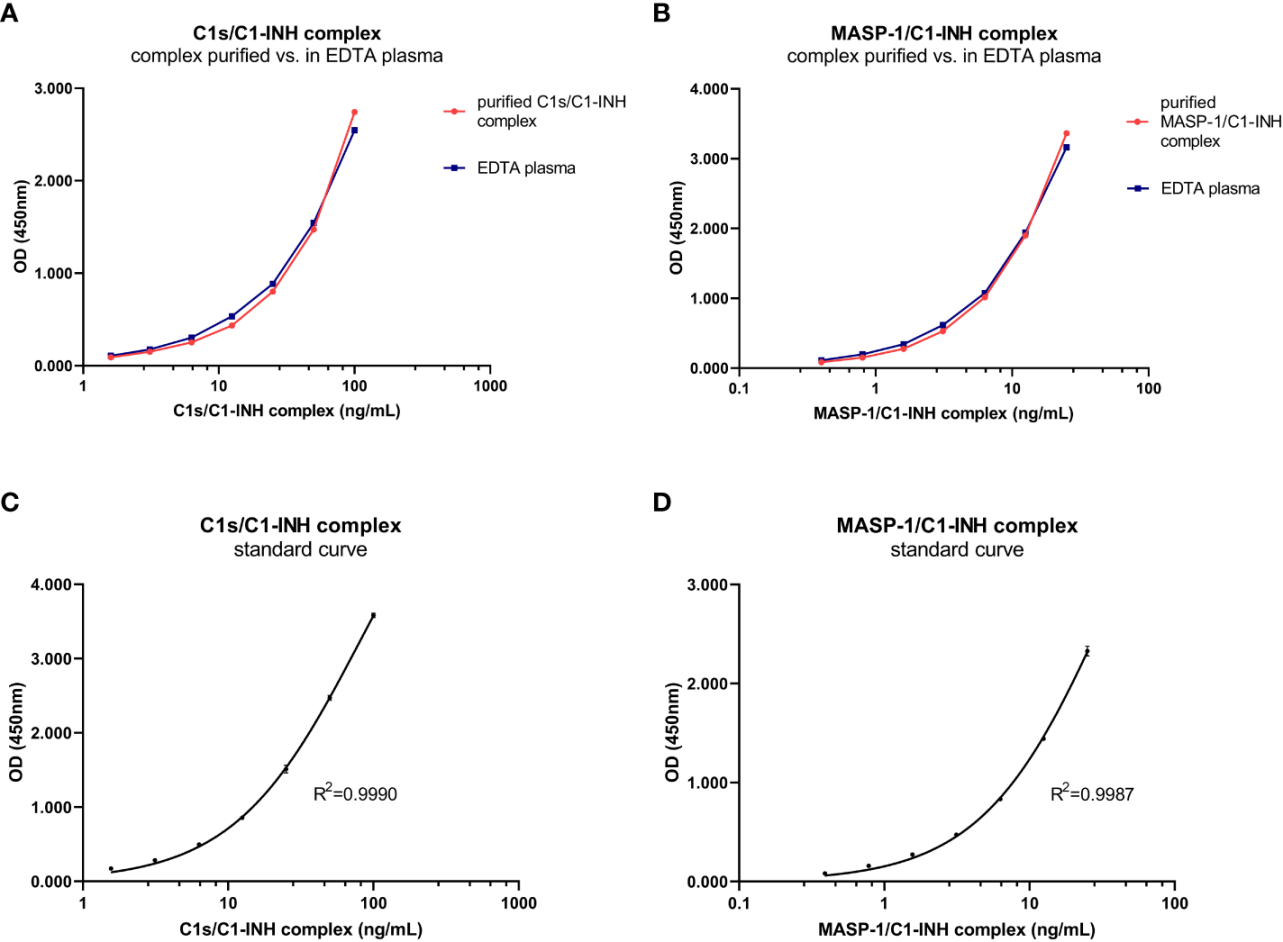
C1-INH complex standards purified in buffer and in EDTA plasma and exemplary standard curves. Parallelism of the C1-INH complexes is shown either purified in buffer (red) or in EDTA plasma (blue) for concentrations ranging from 1.6-100 ng/mL C1s/C1-INH complex **(A)** and 0.4-25 ng/mL MASP-1/C1-INH complex **(B)**. Representative results of a C1s/C1-INH complex standard curve **(C)**, ranging from 0 to 100 ng/mL; and a MASP-1/C1-INH complex standard curve **(D)**, ranging from 0 to 25 ng/mL. The coefficient of determination was obtained using non-linear regression (One-site binding, Hyperbola). OD, optical density; R^2^, coefficient of determination; nm, nanometer; EDTA, Ethylenediaminetetraacetic acid.

The new assays are sandwich ELISAs that use murine monoclonal antibodies specifically recognizing either C1s (C1s/C1-INH assay) or MASP-1 (MASP-1/C1-INH assay), and C1-INH, ensuring that only C1-INH complexes are detected. While the initial assay setup was established using purified C1s/C1-INH and MASP-1/C1-INH complexes as a standard, minimally different curve progressions are obtained when the complexes are measured in EDTA plasma (**Figures 2A, B**). Hence, an EDTA plasma pool with known C1-INH complex concentrations was chosen as a calibrator for both assays, while exemplary standard curves are shown in **Figures 2C, D**. For the C1s/C1-INH assay, the curve progression was linear between 1.6-100.0 ng/mL (LLoQ: 0.2 ng/mL, ULoQ: 100.0 ng/mL) and resulted in a coefficient of determination of R^2^= 0.9990 between measured optical density (OD) values at 450nm and C1s/C1-INH complex concentration. For the MASP-1/C1-INH assay, the curve progression was linear between 0.4-25.0 ng/mL (LLoQ: 0.06 ng/mL, ULoQ: 50 ng/mL), resulting in R^2^ = 0.9987. Non-linear regression (one-site binding, Hyperbola) was chosen for curve fitting. When levels of the respective C1-INH complexes are higher than the highest concentrations of the standard curves, a matrix effect and saturation of the standard curve can be observed (data not shown). Such interactions can be avoided by measuring samples at increased dilutions.

**Figure 3:**
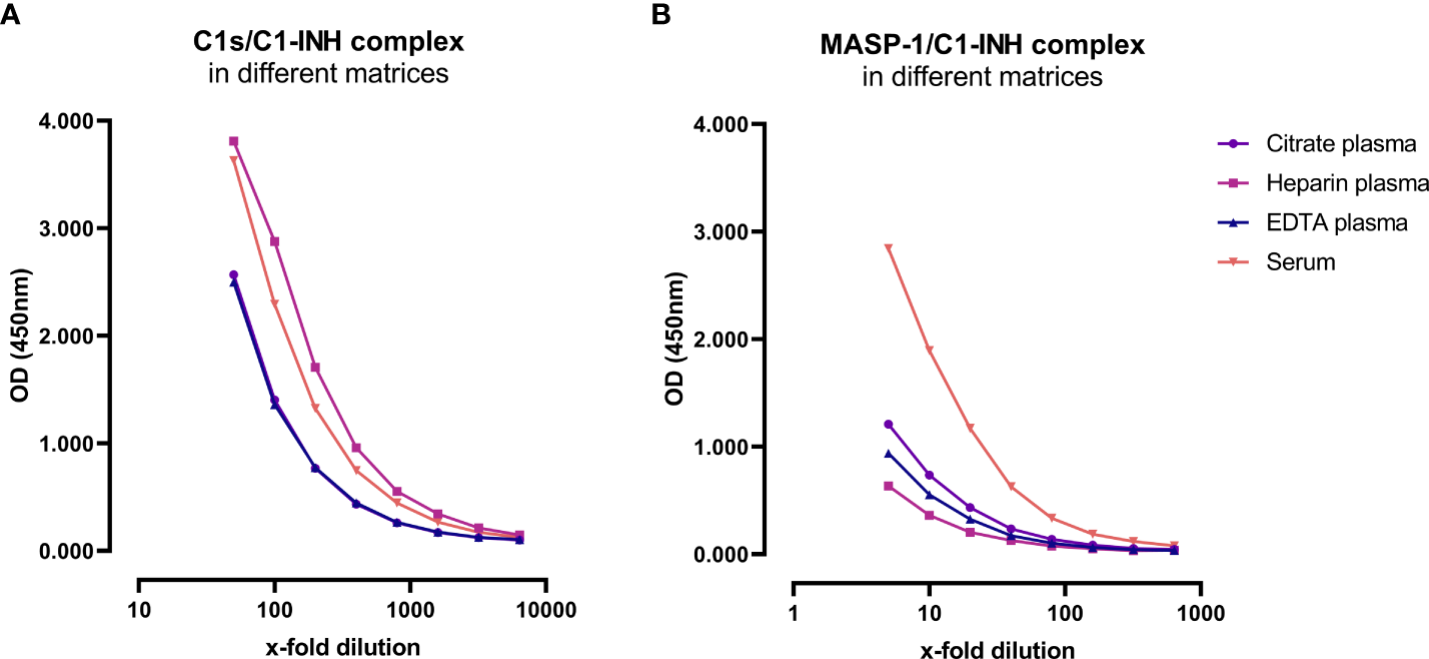
C1-INH complex concentrations in different matrices. Exemplary results of Citrate plasma (purple), Heparin plasma (pink), EDTA plasma (blue) and serum samples (red) from a single individual are plotted in a dilution range from 50x-6400x for the C1s/C1-INH complex **(A)** and in a dilution range from 5x-640x for the MASP-1/C1-INH complex **(B)**. Influence of matrices on C1-INH complex concentrations was investigated in two individuals, while results of one individual each are shown here as an example. OD, optical density; EDTA, Ethylenediaminetetraacetic acid; nm, nanometer.

Matrix effects can occur when a target analyte interacts with matrix components in plasma or serum samples, and this might result in erroneous sample readings. In order to investigate whether matrix effects occur and to evaluate which matrices are suitable for reliable measurements of C1-INH complexes using these new immunoassays, serially diluted citrate plasma, heparin plasma, EDTA plasma and serum samples derived from the same individual were analyzed (**Figure 3**). Calculated concentrations and coefficients of variation (%CVs) for these matrices are listed in **Table 1**. While C1-INH complex concentrations could be measured in all four matrices, the resulting values vary depending on the sample type. C1s/C1-INH complex levels were similar in EDTA and citrate plasma, while higher levels were observed in heparin plasma and serum. Therefore, the latter sample types need to be diluted more during measurements in the ELISA. For the MASP-1/C1-INH complex, highest levels were measured in serum and lower complex levels in all plasma types tested. Coefficients of variation for serum samples were slightly higher compared to plasma samples for both complexes, but all %CVs were below 10% and hence in an acceptable range.

**Table 1:** Analysis of different matrices.

	C1s/C1-INH complex				MASP-1/C1-INH complex			
Matrix	Citrate	Heparin	EDTA	Serum	Citrate	Heparin	EDTA	Serum
**mean concentration(ng/mL)**	2270	6215	2348	4612	37.5	16.6	26.5	121.7
**SD (ng/mL)**	81	279	93	251	1.8	0.8	1.6	10.6
**CV (%)**	3.6	4.5	4.0	5.4	4.7	4.7	5.9	8.7

Representative results of a single individual are listed, while influence of matrices on C1-INH complex concentrations was investigated in two individuals each. Samples were 2-fold serially diluted to establish best dilution ranges over at least three dilution steps for each matrix tested. %CV was calculated to determine the variability for each serial dilution and a %CV ≤20 was considered as minimal matrix effect. SD, standard deviation; %CV, coefficient of variation; EDTA, ethylenediaminetetraacetic acid.

**Figure 4:**
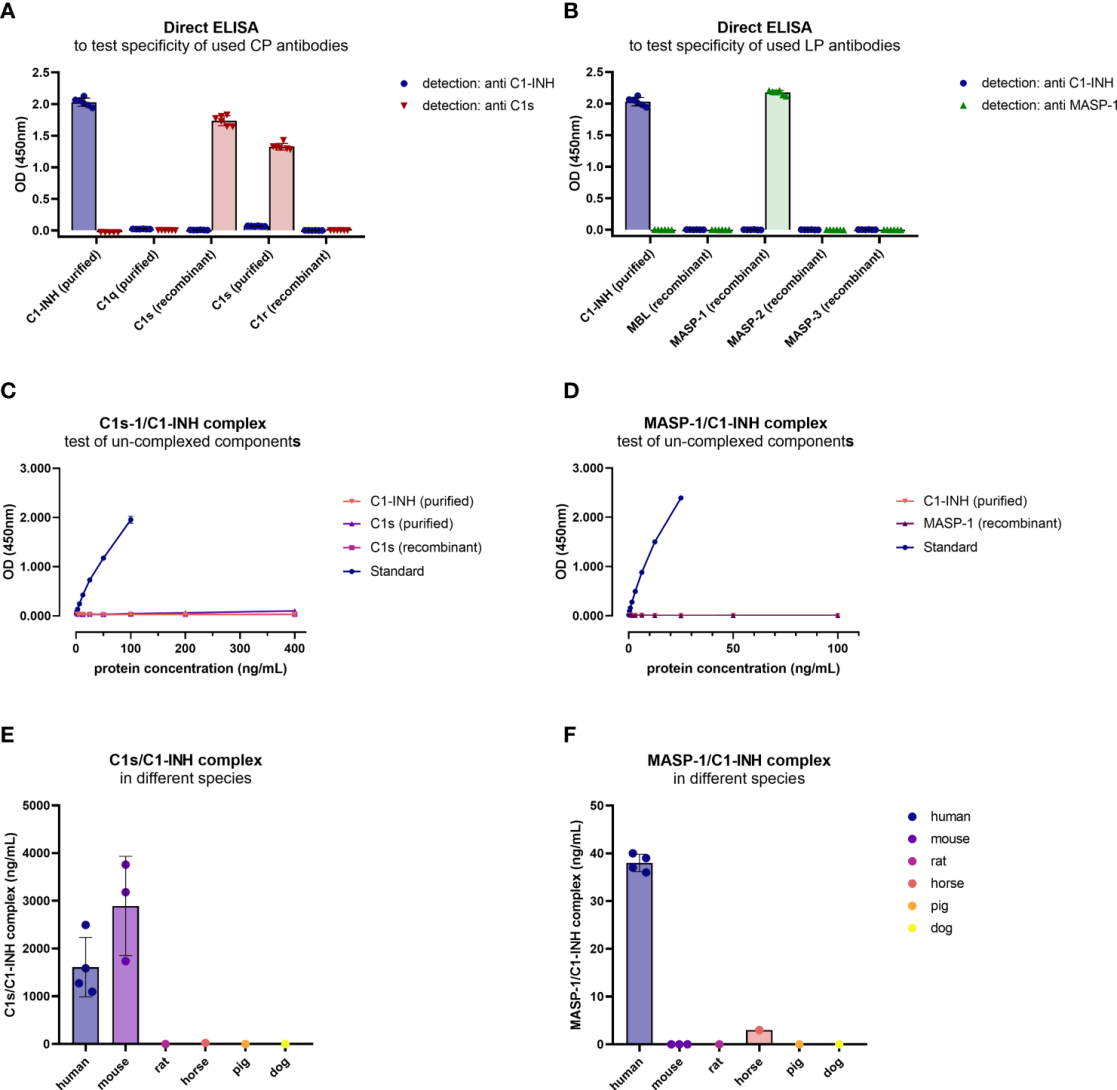
Cross-reactivity of antibodies and new immunoassays. **(A, B)** Binding of antibodies to proteins of the respective pathways was tested in a direct ELISA. For classical pathway proteins, detection was done with either the C1-INH or C1s antibody, while for lectin pathway proteins detection was done with the C1-INH and MASP-1 antibodies also used during assay development. Conditions were tested in duplicates, while the experiment was performed in triplicates. **(C, D)** Cross-reactivity of un-complexed complement components was tested using either purified or recombinant proteins of the complex, dependent on availability. While C1s (purified and recombinant) and C1-INH (purified) were tested in the C1s/C1-INH complex assay **(C)**, MASP-1 (recombinant) and C1-INH (purified) were measured in the MASP-1/C1-INH assay **(D)**. **(E, F)** Cross-reactivity of serum samples from animal origin was investigated in the assays using human EDTA plasma samples (n=4) as a reference, murine serum (n=3), rat serum (n=1), horse serum (n=1), pig serum (n=1) and dog serum (n=1) (**E**: C1s/C1-INH complex, **F**: MASP-1/C1-INH complex). Animal samples were measured 10x less diluted compared to human samples. OD, optical density; nm, nanometer.

Although *in silico* amino acid searches did not identify any proteins with >60% similarity to the antigens used for antibody development, cross-reactivity of the individual antibodies was tested in a direct ELISA using either purified or recombinant proteins of the respective pathways, depending on availability. The anti C1-INH monoclonal antibody showed a strong signal for purified C1-INH, while very weak signals could also be observed detecting purified C1q or C1s (OD450nm<0.1 for both proteins, and hence below the background threshold of OD450nm<0.2 and neglectable) (**Figure 4A**). The antibody did not bind to any of the recombinant proteins tested. The anti C1s antibody showed strong signals for both, recombinant and purified C1s, but not for any other protein tested, while the anti MASP-1 monoclonal antibody only showed signals for recombinant MASP-1 (**Figure 4B**).

After the sandwich ELISA protocol was established, cross-reactivity of the assays was investigated using uncomplexed complement components. When applying both the uncomplexed proteins and the C1-INH complexes (standard curve) to the assays, in concentrations 4x higher compared to the standard curve, only the C1-INH complexes are showing specific signals (**Figures 4C, D**).

Cross-reactivity of the complex assays with other species was tested using blood samples from animal origin. When measuring blood samples from animals (mouse, rat, horse, pig, dog) in the immunoassays, only murine samples (n=3) showed strong cross-reactivity in the C1s/C1-INH assay, while no signals were observed using blood from other animals tested (**Figure 4E**). None of the animal sera tested showed a strong signal in the MASP-1/C1-INH assay (**Figure 4F**).

In order to assess the accuracy of the assays, inter-assay variation (variation of multiple measurements of single samples in a single test run) and intra-assay variation (variation of multiple determinations of a single sample in several test runs performed by different operators) were investigated. Both assays showed inter- and intra-assay coefficients of variation<10%, indicating only low variation between multiple runs as well as between different operators (**Table 2**).

**Table 2:** Inter- and Intra-assay variation of C1-INH complex assays.

C1s/C1-INH complex		sample 1	sample 2	sample 3	sample 4
**Intra-assay variation**					
**Operator 1**	mean aliquot 1-3 (ng/mL)	1161	1967	1595	1285
	SD (ng/mL)	90	121	49	63
	CV (%)	7.8	6.2	3.1	4.9
**Operator 2**	mean aliquot 1-3 (ng/mL)	1245	2135	1868	1399
	SD (ng/mL)	47	166	99	43
	CV (%)	3.8	7.8	5.3	3.1
**Inter-assay variation**					
	mean Operator 1 and 2 (ng/mL)	1203	2051	1731	1342
	SD (ng/mL)	79	159	165	79
	CV (%)	6.6	7.8	9.5	5.9
MASP-1/C1-INH complex		sample 1	sample 2	sample 3	sample 4
**Intra-assay variation**					
**Operator 1**	mean aliquot 1-3 (ng/mL)	273.2	115.5	57.3	35.5
	SD (ng/mL)	6.6	0.7	3.6	0.5
	CV (%)	2.4	0.6	6.2	1.5
**Operator 2**	mean aliquot 1-3 (ng/mL)	259.0	108.3	52.4	34.7
	SD (ng/mL)	8.1	2.4	1.2	0.6
	CV (%)	3.1	2.2	2.4	1.8
**Inter-assay variation**					
	mean Operator 1 and 2 (ng/mL)	266.1	111.9	54.9	35.1
	SD (ng/mL)	10.2	4.3	3.6	0.7
	CV (%)	3.8	3.8	6.6	1.9

Intra-assay variation (multiple determinations of single samples within a single test run) and inter-assay variation (multiple determinations of single samples in several assay runs) was determined for both complex assays by calculation of mean concentrations and %CV using 3 independent aliquots of 4 different samples in 2 test runs (performed by 2 different operators). A %CV ≤10 indicates low variation for the intra-assay variation, while a CV% ≤20 indicates low inter-assay variation. %CV, coefficient of variation.

**Figure 5:**
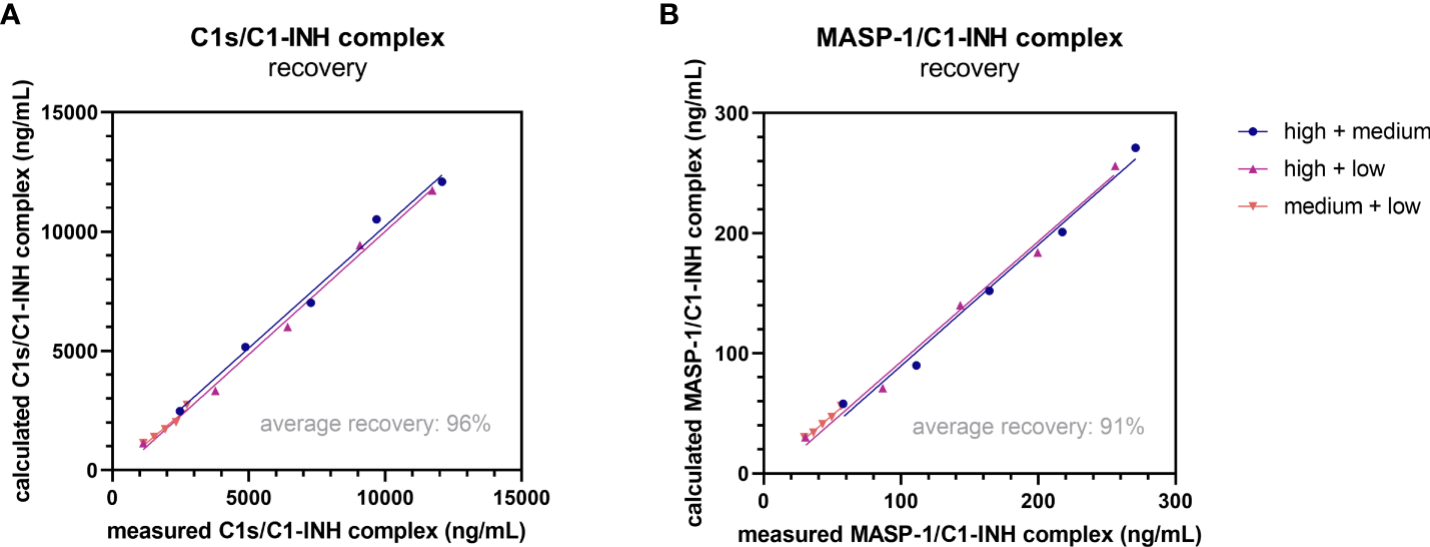
Recovery of complexes in EDTA plasma. Recovery of the C1s/C1-INH complex **(A)** and MASP-1/C1-INH complex **(B)** was investigated by mixing EDTA plasma samples of three individuals with low, middle and high complex concentration in different ratios (100 + 0, 75 + 25, 50 + 50, 25 + 75, 0 + 100). After 30 min of incubation, C1-INH complex concentrations were determined in the respective assays and recovery (difference between expected and observed C1-INH complex concentration) was calculated according to the equation stated in the Material and Methods section. EDTA, ethylenediaminetetraacetic acid; min, minutes.

Recovery of the C1-INH complexes was analyzed in EDTA plasma by mixing of samples with varying complex concentrations in different ratios. An average recovery >90% was seen for both immunoassays (**Table 3**). In general, a recovery between 80-120% was accepted in the experiment, indicating high accuracy. The expected concentrations of the single samples are plotted against the measured concentrations in **Figure 5**. In both cases expected vs. measured concentration correlated significantly (p<0.0001), with correlation coefficients of R=1.000 for the C1s/C1-INH complex and R=0.999 for the MASP-1/C1-INH complex.

**Table 3:** Recovery of C1s/C1-INH and MASP-1/C1-INH complexes in EDTA plasma.

	Sample composition (%)						
	100 + 0	75 + 25	50 + 50		25 + 75		0 + 100
	mixture of sample 1 (high) and sample 2 (medium)						
C1s/C1-INH (ng/mL)	12086	10517	7014		5160		2476
Recovery (%)	–	109	96		106		–
	mixture of sample 1 (high) and sample 3 (low)						
C1s/C1-INH (ng/mL)	11728	9436	6002		3325		1135
Recovery (%)	–	104	93		88		–
	mixture of sample 2 (medium) and sample 3 (low)						
C1s/C1-INH (ng/mL)	2736	2013	1712		1386		1136
Recovery (%)	–	86	88		90		–
	mixture of sample 1 (high) and sample 2 (medium)						
MASP-1/C1-INH (ng/mL)	271	201	152		90		58
Recovery (%)	–	92	93		81		–
	mixture of sample 1 (high) and sample 3 (low)						
MASP-1/C1-INH (ng/mL)	256	184	140		71		30
Recovery (%)	–	92	97		82		–
	mixture of sample 2 (medium) and sample 3 (low)						
MASP-1/C1-INH (ng/mL)	56	47	41		34		30
Recovery (%)	–	95	96		94		–

Recovery of the C1s/C1-INH complex (upper part) and MASP-1/C1-INH complex (lower part) was investigated by mixing EDTA plasma samples of three individuals with low, middle and high complex concentration in different ratios (100 + 0, 75 + 25, 50 + 50, 25 + 75, 0 + 100). After 30min. of incubation, C1-INH complex concentrations were determined in the respective assays and recovery (difference between expected and observed C1-INH complex concentration) was calculated according to the equation stated in the Material and Methods section. EDTA, ethylenediaminetetraacetic acid; min, minutes.

In summary, the tests performed during assay development and optimization showed that both assays are able to measure the C1-INH complexes in an accurate, specific and reliable manner. Described assay characteristics are summarized in **Table 4**.

**Table 4:** Summarized characteristics of newly developed C1-INH complex assays.

	C1s/C1-INH complex assay	MASP-1/C1-INH complex assay
**Standard range**	100.0 – 1.6 ng/mL	25.0 – 0.4 ng/mL
**Limit of quantification** **ULoQ** **LLoQ**	100.0 ng/mL 0.2 ng/mL	50 ng/mL 0.06 ng/mL
**cross-reactivity with uncomplexed components**	no	no
**cross-reactivity with animal serum** **(mouse, rat, horse, pig, dog)**	mouse	no
**Inter-assay variation**	< 10%	< 10%
**Intra-assay variation**	< 10%	< 10%
**Matrices to be used** **(minimal required dilution)**	EDTA plasma (100x) Citrate plasma (100x) Heparin plasma (200x) Serum (200x)	EDTA plasma (5x) Citrate plasma (5x) Heparin plasma (5x) Serum (10x)
**Measured complex concentration in healthy individuals, n=96**	1846 ± 1060 ng/mL [mean ± 2SD]	40.5 (13.8-87.9) ng/mL [median (2.5-97.5 percentile range)]

EDTA, ethylenediaminetetraacetic acid; ULoQ, upper limit of quantification; LLoQ, lower limit of quantification; SD, standard deviation.

### Validation of C1-INH complexes as markers for early complement activation markers

**Figure 6:**
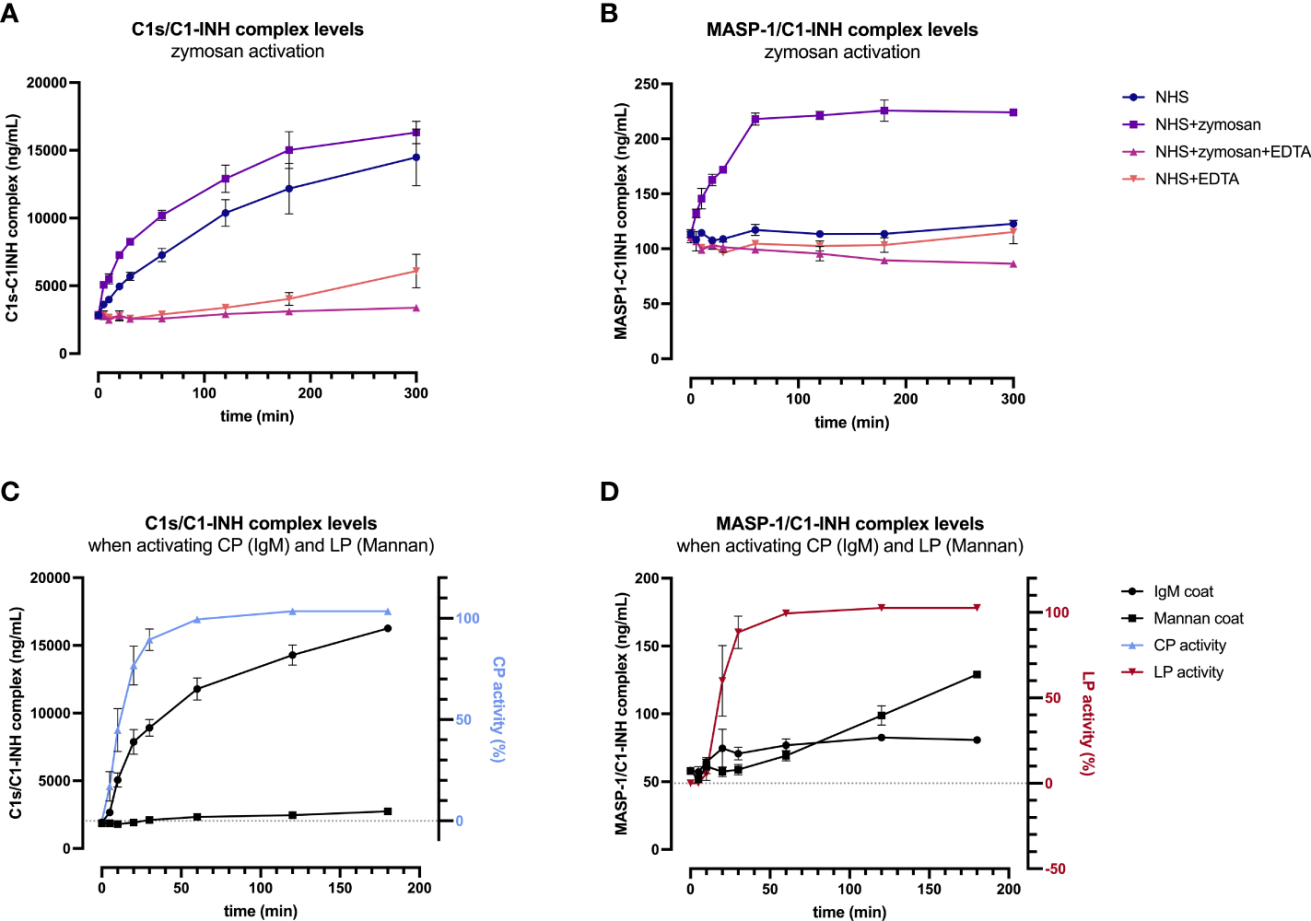
Validation of C1-INH complexes as activation markers: zymosan-initiated **(A, B)** and pathway-specific **(C, D)** complex formation over time. For zymosan-activation **(A, B)**, normal human serum was incubated either alone or with zymosan, EDTA, or a mix thereof, for up to 5 hours at 37 °C. Samples were taken at several time points and concentrations of C1s/C1-INH complex **(A)** and MASP-1/C1-INH complex **(B)** were determined using the new immunoassays. Plotted values show C1-INH complex concentrations (mean ± SD) of two independent activation experiments performed in duplicates each, while effects of added compounds (zymosan, EDTA) and incubation time on C1-INH complex levels were analyzed with a two-way ANOVA with Tukey’s multiple comparisons test. For the C1s/C1-INH complex, a statistically significant effect was seen for the zymosan-treated sample for both, the incubation time (p<0.0001) as well as the treatment (NHS+zymosan vs. NHS: p=0.0399, NHS+zymosan vs. NHS+zymosan+EDTA: p<0.0001, NHS+zymosan vs. NHS+EDTA: p<0.0001). For the MASP-1/C1-INH complex, the zymosan-treated sample did also show statistically significant effects for the incubation time (p<0.0001) as well as the treatment (NHS+zymosan vs. all other treatments tested: p<0.0001). For pathway-specific activation **(C, D)**, normal human serum was incubated on wells coated with either IgM (CP) or mannan (LP), for up to 3 hours at 37 °C. After incubation, C1-INH complex levels were determined in the supernatant (**C**: C1s/C1-INH complex; **D**: MASP-1/C1-INH complex), while C9 neoepitope formation was measured on the respective wells (WIESLAB^®^ Complement System kits). Plotted values show mean concentrations of C1-INH complexes (mean ± SD) and normalized CP and LP activity (as measured by C9 neoepitope formation using positive and negative controls provided in the kits) of three independent experiments. NHS, normal human serum; EDTA, ethylenediaminetetraaceticacid; SD, standard deviation; min, minutes; IgM, immunoglobulin M; TCC, terminal complement complex.

In order to validate the C1-INH complexes as markers for early classical and early lectin pathway activation, normal human serum (complement-preserved) was activated by zymosan and changes of C1s/C1-INH complex and MASP-1/C1-INH complex levels over time were measured after activation using the newly developed immunoassays. The results showed a strong increase in the complex concentrations for both C1-INH complexes investigated, while the concentrations at time zero (T0) were comparable for all four approaches tested, with mean concentrations of 2829 ± 138 ng/mL C1s/C1-INH complex and 111.4 ± 3.5 ng/mL MASP-1/C1-INH complex (**Figures 6A, B**).

For the C1s/C1-INH complex, complex levels in the NHS+zymosan sample increased reaching a plateau of around 16000 ng/mL after 5 h of activation, a 5.7-fold increase compared to the starting concentration (**Figure 6A**). Complex concentrations in the NHS sample without any additions also increased up to a level of 14000 ng/mL C1s/C1-INH within 5 h, indicating strong auto-activation of the classical pathway when no EDTA is present. Both controls with EDTA were stable for up to 2 h, while in the NHS+EDTA sample an increase in C1s/C1-INH complexes up to 5000 ng/mL was measured when incubating for more than 2 hours at 37°C.

Formation of the MASP-1/C1-INH complex seems to reach its maximum more rapid, with the highest rate of C1-INH complex formation again being observed in the NHS sample activated with zymosan (**Figure 6B**). Here the complex concentration doubled to 224 ng/mL within one hour, while the concentration did not increase further when activation is continued out to 5 h. In contrast to the C1s/C1-INH complex, no significant increase in MASP-1/C1-INH complex was seen in the normal human serum without activator (zymosan) or in the samples additionally containing EDTA (NHS+EDTA and NSH+zymosan+EDTA).

A two-way ANOVA showed highly significant effects of the added compounds (EDTA or zymosan) as well as the incubation time for both complexes, when compared to the control samples (results in legend of **Figure 6**).

In addition, formation of C1-INH complexes was also investigated in conditions where only one of the two pathways were specifically activated. When only activating the classical pathway using IgM coating, concentrations of C1s/C1-INH complex did increase in accordance with CP activity, measured by the formation of C9 neoepitope within the same samples, while MASP-1/C1-INH complex levels did not change markedly (**Figures 6C, D**). During CP activation, C1s/C1-INH complex levels already show a significant increase within 5 min (p=0.0101), and this increase stayed highly significant when activating for 10 minutes or longer, when compared to baseline (p>0.0001). Vice versa, when specifically activating the lectin pathway *via* mannan coating, there is a significant increase in MASP-1/C1-INH complex levels (60 min activation: p=0.0140; >60 min activation: p<0.0001) as well as of the LP activity (again measured by C9 neoepitope formation) over time, whereas C1s/C1-INH complex concentrations remained unchanged when compared to baseline values (**Figure 6D**). Only when activating the LP for more than 2h at room temperature, C1s/C1-INH complex levels also differ significantly from baseline (p=0.0456), which can be explained by auto-activation of the classical pathway. Those findings further confirm the potential of C1-INH complexes as specific markers for early classical and early lectin pathway activation.

**Figure 7:**
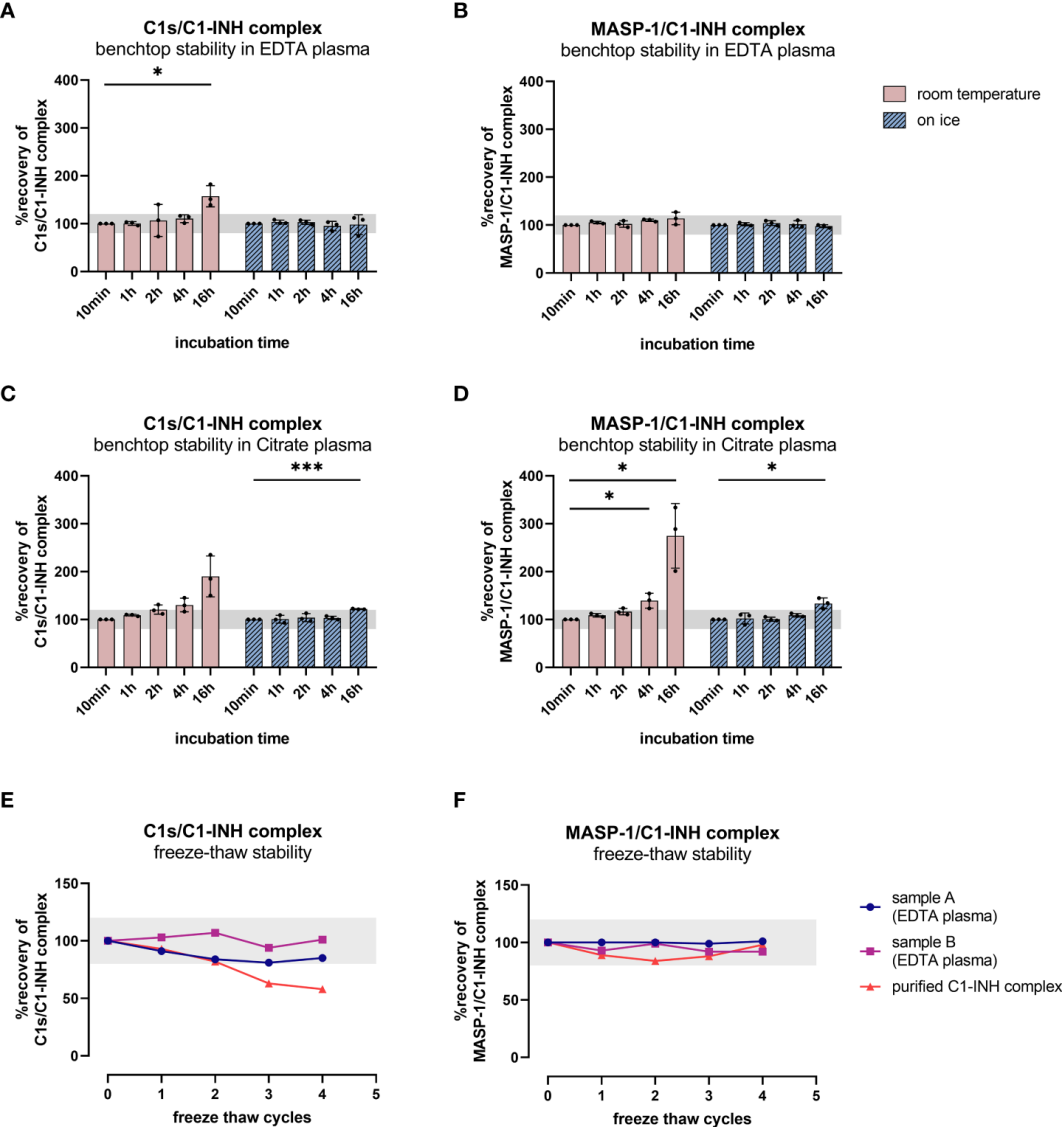
Benchtop stability of C1-INH complexes in EDTA plasma and Citrate plasma **(A–D)** and freeze-thaw stability of complexes in EDTA plasma and in purified form **(E, F)**. For benchtop testing (panels **A–D**), samples were stored at room temperature (red graphs) or on ice (blue graphs) for different time intervals (10 min-16 h). Afterwards concentrations of either C1s/C1-INH complex (**A**: EDTA plasma, **C**: Citrate plasma) or MASP-1/C1-INH complex (**B**: EDTA plasma, **D**: Citrate plasma) were measured in the samples. C1-INH concentrations of the 10 min samples were set as 100% and the amount of complexes at other time points was calculated in relation to the 10 min sample. Differences to the 10 min sample were calculated using one sample t-test (* p<0.05, *** p<0.001, non-significant results are not marked). Due to natural variation in biomarker measurements, acceptable concentrations ranged from 80-120% compared to the concentration in the respective 10 min sample. For analysis of freeze-thaw stability (panels **E, F**), aliquots of either purified C1-INH complex (plotted in red) or two independent EDTA plasma samples (plotted in blue and pink) were exposed to 0 to 4 freeze-thaw cycles and concentrations of C1s/C1-INH complex **(E)** and MASP-1/C1-INH complex **(F)** were determined in the respective sandwich ELISAs. Due to natural variation when measuring biomarkers, acceptable complex concentrations ranged from 80-120% of the original concentration (0 freeze thaw cycles) and the accepted range is marked in grey on the figures. min, minutes; h, hours; EDTA, ethylenediaminetetraacetic acid.

Since increasing C1-INH complex levels were also observed without the addition of zymosan, the rate of auto-activation of the respective complement pathways was investigated in EDTA plasma and citrate plasma by incubating several samples for up to 16 h at room temperature and on ice. While incubation on ice for up to 16 h does not strongly affect C1-INH complex concentrations in EDTA plasma, an increase is seen when samples are kept at room temperature for more than four hours (**Figures 7A–D**). Especially for the C1s/C1-INH complex, additional complex formation is observed even in EDTA plasma at room temperature when incubating for more than two hours (**Figure 7A**).

Citrate plasma showed additional complex formation if samples are kept at room temperature for more than 1 h (**Figures 7C, D**), while concentrations are more than double the initial levels after 16 h at room temperature. When incubating on ice, the formation of new C1-INH complexes occurs more slowly, but again at a higher rate than when compared to EDTA plasma.

Those findings indicate the importance of sample handling and the usage of EDTA plasma when investigating complement activation products in general, but more specifically C1-INH complexes.

To obtain more information about appropriate sample handling, we also investigated freeze-thaw stability of the C1-INH complexes in EDTA plasma samples as well as in purified form (commercially available C1s/C1-INH complex and in-house MASP-1/C1-INH complex). Exposure of the samples to up to four freeze-thaw cycles revealed that both complexes are relatively stable in EDTA plasma for multiple rounds of freezing and thawing. However, the purified C1s/C1-INH complex seems to be more prone to degradation with an increasing number of freeze-thaw cycles compared to the MASP-1/C1-INH complex (**Figures 7E, F**).

### Clinical validation of the novel immunoassays in healthy controls and COVID-19

The newly developed assays were used to measure C1s/C1-INH complex and MASP-1/C1-INH complex levels in healthy individuals (n=96) as well as in a total of 414 COVID-19 patients.

**Figure 8:**
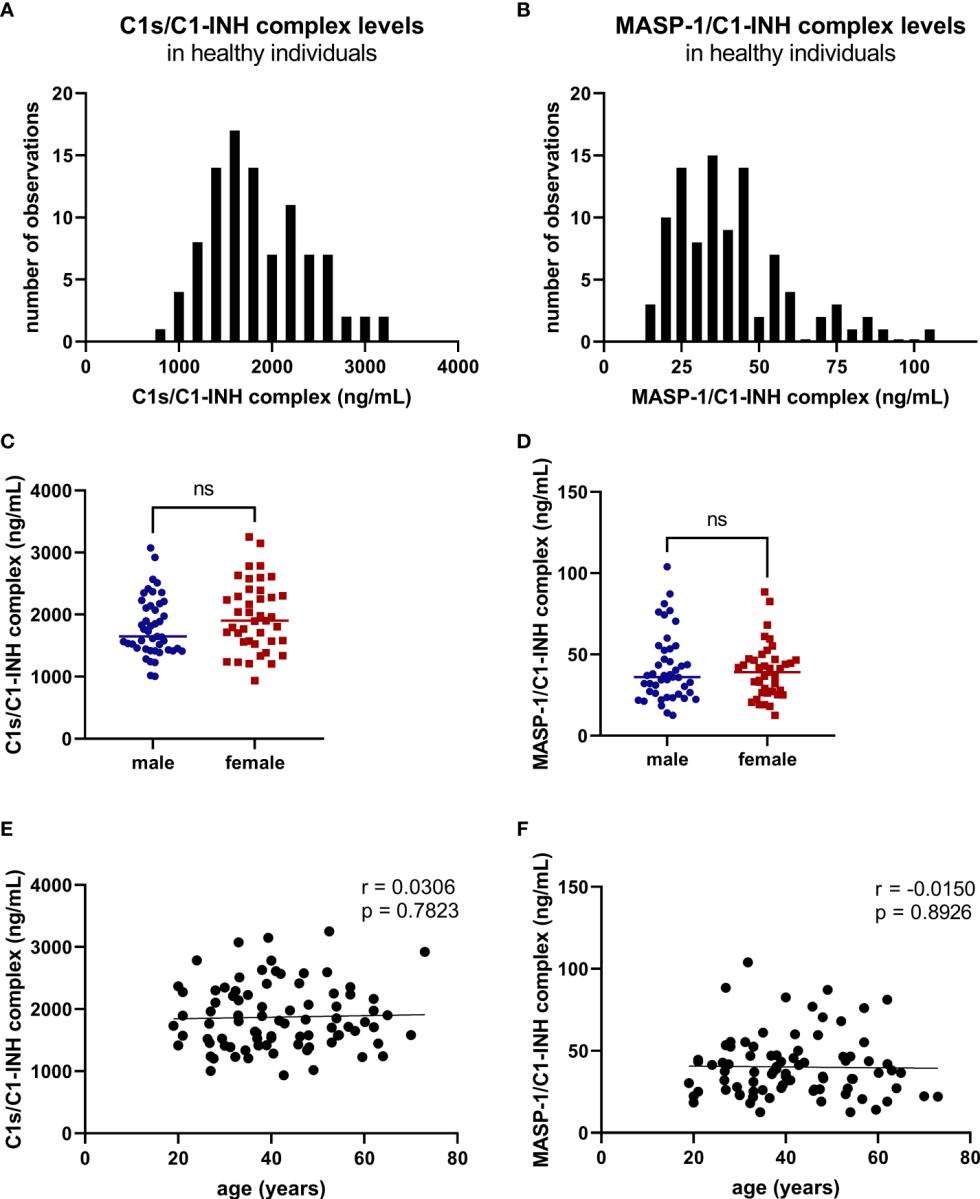
C1-INH complex levels in healthy adult individuals. C1-INH complex concentrations were measured in 96 healthy adult individuals, using the new immunoassays. Distributions of C1s/C1-INH complex **(A)** and MASP-1/C1-INH complex concentrations **(B)** are shown in histograms. Concentrations in healthy adults were furthermore stratified according to gender (**C**: C1s/C1-INH complex, **D**: MASP-1/C1-INH complex) and age (**E**: C1s/C1-INH complex, **F**: MASP-1/C1-INH complex). No significant differences between males (n=50) and females (n=46) were observed when comparing the groups using Mann-Whitney test. ns, not significant; r, correlation coefficient.

In healthy individuals, measurement of C1-INH complexes resulted in a mean physiological concentration of 1846 ± 1060 ng/mL C1s/C1-INH complex (mean ± 2SD) and in 36.9 (13.18 - 87.89) ng/mL MASP-1/C1-INH complex [median (2.5 percentile range – 97.5 percentile range)]. Distribution of the concentration levels measured in healthy adults are shown in **Figures 8A, B**. While physiological C1s/C1-INH complex concentrations are normally distributed within the observed concentration range (p=0.0974), MASP-1/C1-INH complex levels are right-skewed (p<0.0001).

Besides that, no significant differences in C1-INH complex levels between healthy males (n=50) and females (n=46) were observed (**Figures 8C, D**). Additionally, no correlation is present between the C1-INH complex levels and the age of healthy adults (**Figures 8E, F**).

In order to perform a proof-of-concept study including diseased samples, the new immunoassays were utilized to measure C1-INH complex concentrations in a cohort of 414 COVID-19 patients (median delay between symptom onset or positive PCR test and sampling: 10.0 days (IQR 6.0 – 27.8)).

**Figure 9:**
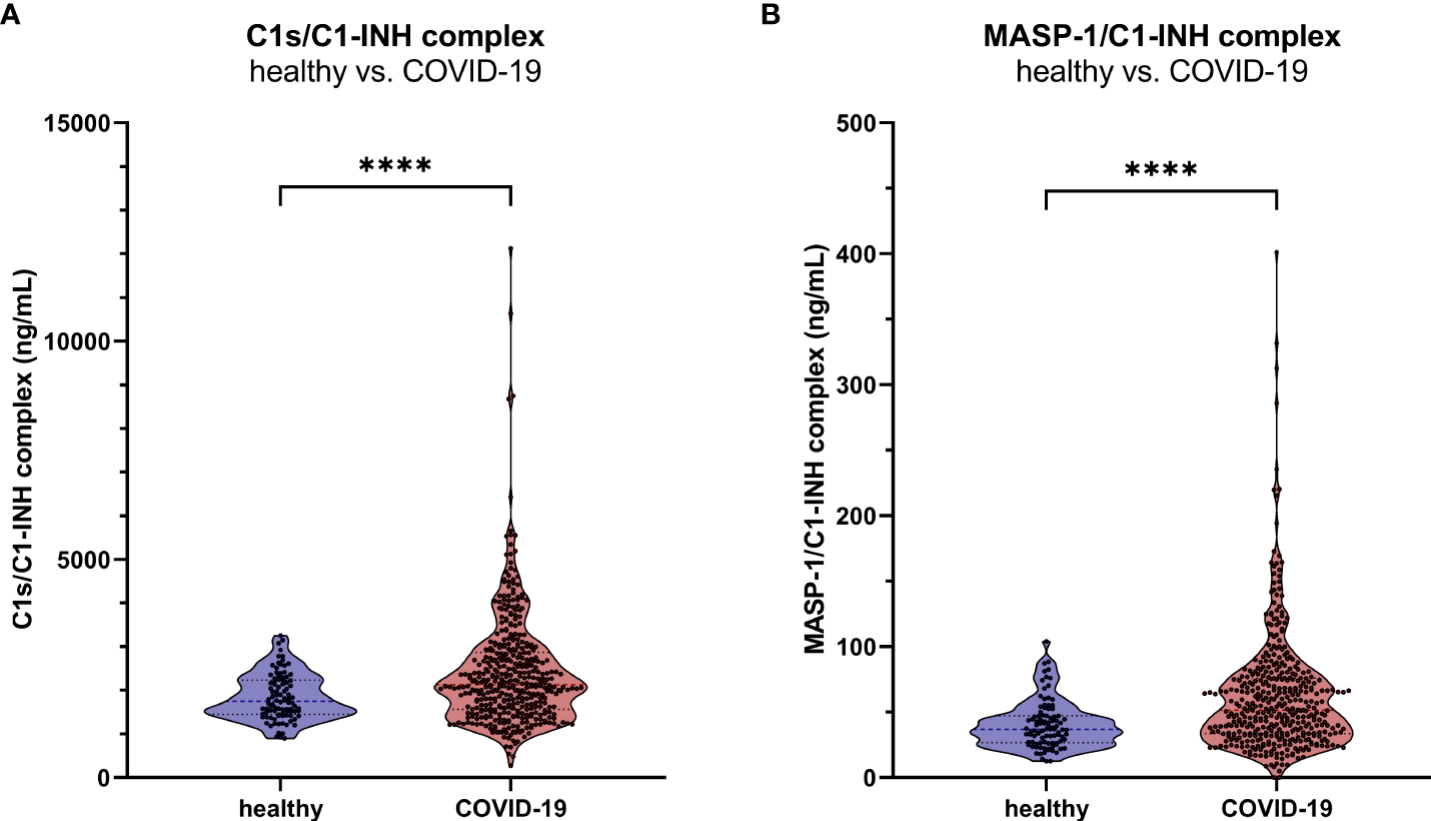
C1-INH complex levels in adult COVID-19 patients. The new immunoassays were clinically validated by the measurement of C1s/C1-INH complex **(A)** and MASP-1/C1-INH complex levels **(B)** in 414 COVID-19 patients as well as in 96 healthy controls. P values for the pair-wise group comparisons (healthy vs. COVID-19) were calculated by the Mann-Whitney test (**** p<0.0001).

Measurements showed increased C1-INH complex levels in COVID-19 patients when compared to healthy controls, with a mean concentration of 2407 ± 1283 ng/mL C1s/C1-INH complex (Mann-Whitney U test: p<0.0001, **Figure 9A**) and 51.5 (33.5-76.1) ng/mL MASP-1/C1-INH complex (Mann-Whitney U test: p<0.0001, **Figure 9B**).

## Discussion

Although complement dysregulation and overactivation underlies many pathological conditions (40–42), its precise role is often not clear. To understand how complement is dysregulated in a certain type of disease, it is important to unravel which pathway (either the CP, LP or AP) is (over)activated and to what extent. Here we propose that C1-INH complexes might be suitable markers to monitor early classical or lectin pathway activation. However, as tools for accurately measuring these markers are currently lacking, we aimed to develop novel immunoassays that are able to assess these complexes in human samples. We successfully developed two sandwich enzyme-linked immunosorbent assay that allow the quantification of C1s/C1-INH and MASP-1/C1-INH complex levels in a comparable manner in human plasma and serum samples. In addition, we define a reference range in healthy controls for both complexes. Beyond that, we show that measurement of ongoing complement activation is possible through the determination of C1-INH complex levels, where C1s/C1-INH levels indicate early classical pathway activation and MASP-1/C1-INH complex levels serve as a marker for early lectin pathway activation.

The newly developed immunoassays show low inter- and intra assay variation as well as high recovery, indicating that the complexes can be quantified in a reliable and robust manner. The monoclonal antibodies used for both assays are highly specific as no or only very weak cross-reactivity was observed for the un-complexed proteins (C1s, MASP-1 and C1-INH) and related proteins (C1q, C1r, MBL, MASP-2 and -3) in both a direct or sandwich ELISA setup. Although the weak signals for both the un-complexed as well as the related proteins are probably due to a non-specific background signal, it cannot be excluded that some of these signals are caused by contamination from the complexes themselves. When available, we used proteins purified from human blood (C1q, C1s, C1-INH). It is possible that these purified proteins contain traces of the complexes in addition to the individual proteins as purification methods are never 100% successful. Indeed, for the purified proteins that were commercially available, the suppliers only guarantee a purity of ≥90%. Also cross-reactivity with species other than human was tested and the C1s/C1-INH complex assay strongly cross-reacted with murine samples, although not fully validated yet, making it also a potential tool for researchers investigating classical pathway activation or CP related diseases in mice. The MASP-1/C1-INH complex assay did not show strong signals when using samples of animal origin.

Robust immunoassays are particularly important within the complement field, as it has been shown that measuring complement components in a reliable way is challenging. Large variation exists in complement measurements between different laboratories, partly caused by a lack of standardized assays and reagents (43). As well known for other complement measurements (44, 45), sample type and handling is crucial when determining C1-INH complex levels. The assays described here can be used for all human plasma types and serum, while dilutions might have to be adopted depending on the matrix used. However, heparinized plasma should be avoided when measuring C1-INH complexes as low signals for the MASP-1/C1-INH complex were observed when using heparin plasma. This effect might be caused by the fact that MASP-1, in the presence of heparin, is more likely to form complexes with antithrombin than with C1-INH (46, 47). Due to increased complex formation between MASP-1 and antithrombin, the MASP-1/C1-INH complex formation might be diminished. In contrast, C1s/C1-INH complex levels were highest in heparin plasma in our experiments. A possible explanation is that heparin is able to enhance the rate of inhibition of active C1s by C1-INH (48), leading to artificial complex formation.

Additional complex formation can also occur in serum samples, which is especially true for the C1s/C1-INH complex. In general, it is recommended to use EDTA plasma samples when the activation state of complement pathways at a given time-point is investigated. EDTA chelates both Ca^2+^ and Mg^2+^ and thereby blocks the function of the complement by destabilizing the pattern-recognition complexes (49–51). Our results show that an EDTA concentration ≥10 mM was able to effectively minimize *in vitro* complement activation for either 4 h at room temperature or 16 h on ice, also shown by Yang and co-workers in the past (52). Typically, EDTA plasma should be used when investigating C1-INH complex levels, samples should be stored at -80°C until further usage and kept on ice after thawing until the measurements in order to avoid *de novo* formation of C1-INH complexes *ex vivo*. When sample handling is not appropriate, particularly the classical pathway can be activated (53). This spontaneous CP activation without addition of activating reagents such as zymosan can be caused by naturally circulating immune complexes or by Ig aggregation during incubation *ex vivo* (53, 54). The MASP-1/C1-INH complex seems to be less prone to sample handling. As expected, no increase of MASP-1/C1-INH complex levels was observed when incubating NHS without zymosan, since no substance specifically activating the lectin pathway is present in serum under physiological conditions.

During assay development, the stability of the complexes was also investigated after freeze-thawing. C1-INH complexes in purified form are more at risk of degradation than in plasma during repeated freeze-thawing, as we observed that complex concentrations in EDTA plasma remain relatively stable and do not decrease much after up to four freeze-thaw cycles. Similar findings were made earlier for C1rs/C1-INH and other complement activation products (55), allowing reliable determination of C1-INH complexes also in samples that have been thawed before, provided that sample handling in between freezing is appropriate.

After establishing a first prototype assay for both complexes, we further investigated whether these complexes indeed could serve as biomarkers for assessing early CP and/or LP activation. For this, Zymosan A, a carbohydrate polymer prepared from *Saccharomyces cerevisiae* cell walls was used. It is mainly known for AP activation through enhancement of alternative pathway C3 convertase assembly and stability (56), but can also induce LP and CP activation (57, 58). Therefore, Zymosan A was chosen to monitor *in vitro* CP and LP activation. When complement-preserved serum samples were incubated with zymosan, both C1-INH complexes showed an increase in concentration over time. Besides that, specific activation of the classical pathway led to a strong increase only in the levels of C1s/C1-INH complex as well as the formation of C9 neoepitope, while selective activation of the lectin pathway only resulted in formation of MASP-1/C1-INH complexes (**Figure 6**). These results provide evidence that the complexes may specifically differentiate between activation of the early classical or lectin pathway, and are potentially suitable markers for assessing and monitoring early CP and/or LP activation in various *in vitro* and *in vivo* conditions.

So far, the only way to investigate CP and LP activity in general are either hemolytic assays or functional ELISAs. The former are based on hemolysis of antibody-sensitized erythrocytes of animal origin by complement components of the added samples (59), while the latter are determining activity of the different pathways by measuring a neo-epitope generated by the MAC formation following activation (60, 61). Both require complement-preserved serum of the specimen to be tested and do not give an indication about ongoing activation of the respective pathways at a given time-point. Availability of complement-preserved serum is often a problem, especially when it comes to investigations of big cohorts. Measurement of the C1-INH complexes as described here does not require complement-preserved samples and can also be done in a more standardized way compared to hemolytic assays relying on fresh animal blood cells for every experiment.

The reference range of C1s/C1-INH complex in healthy adults was found to be 1846 ± 1060 ng/mL (mean ± 2SD), and hence is in line with concentrations already published in the literature utilizing in-house immunoassays. C1s/C1-INH complex concentrations previously reported were 1.0 ± 0.2 mg/L in plasma and 2.1 ± 0.8 mg/L in serum of nine healthy individuals (62), or 25.04 (13.9-36.9) nM in EDTA plasma of six healthy controls (30). While the C1s/C1-INH complex concentration was normally distributed, MASP-1/C1-INH complex levels were slightly right-skewed in healthy adults and the distribution did not pass normality testing. This indicates that other factors may have an effect on MASP-1/C1-INH complex levels, like involvement of the respective proteins in other physiological processes or influence of the genetic background of the individuals (63). However, further investigations are necessary in order to confirm potential causes of the observed right-skewed distribution. Due to a missing normally distribution, the 2.5 - 97.5 percentile range was used to define a reference range for the new lectin pathway activation marker, resulting in a physiological concentration of 36.9 (13.18 -87.89) ng/mL MASP-1/C1-INH complex. Although differences in some complement protein levels are reported to be significantly influenced by gender and age (64), no significant changes in C1-INH complex levels between males and females were observed in our study, which is in line with previous investigations of several complement activation products, also including C1rs/C1-INH (55). Besides that, the complex levels did not correlate with age in healthy adults, so probably no correction for age or gender is necessary in future studies investigating C1-INH complex levels. Additional studies in other cohorts are needed to determine whether these ranges are broadly applicable.

Finally, we showed increased C1-INH complex levels in patients with COVID-19 when compared to healthy controls. In COVID-19, complement is known to be activated *via* all three pathways. While lectin pathway activation occurs through direct binding of MBL to viral envelope particles (65, 66), the classical pathway is activated later through recognition of circulating SARS-CoV-2 specific antibodies by C1q (67, 68) or through immune complexes with IgG bound to proteins of the virus (69). Besides the positive feedback loop function of the alternative pathway upon CP and LP activation, competition of SARS-CoV-2 with factor H for binding sites at heparan sulfate can further increase AP activation (67, 70). Since complement is known to be activated in COVID-19 (34, 35), this cohort was chosen as a technical cohort to see whether C1-INH complex levels differ between healthy and diseased individuals. Measurement of C1-INH complexes confirms significantly higher complex levels in COVID-19 compared to healthy controls, indicating ongoing early activation of both, the classical and lectin pathway [median delay between symptom onset or positive PCR test and sampling: 10.0 days (IQR 6.0 – 27.8)]. Further investigations in this direction are necessary, especially when it comes to different severity groups or disease outcome, correlations with other complement components or potential triggers of the respective pathways, such as associations between C1s/C1-INH levels and anti SARS-CoV-2 antibody concentrations or MASP-1/C1-INH complex concentrations and lectin pathway pattern recognition molecule levels.

However, the findings are in line with measurements using in-house methods for C1-INH complex determinations in other conditions where complement is involved in the disease course. As an example, Füst et al. investigated C1rs/C1-INH complex levels in a cohort of HIV patients as a measure of classical pathway activation. In this study, 1.5 times higher complex levels were measured in HIV seropositive patients compared to seronegative patients and healthy controls (71). Besides that, increased C1-INH complex concentrations were also found in previous publications investigating C1s/C1-INH complex levels as an indicator for classical pathway activation in RA and SLE patients (32) or (C1-INH)_2_ C1r-C1s complexes to indicate C1 activation in glomerulonephritis (31). So far, there are no publications discussing the MASP-1/C1-INH complex as a potential marker for early lectin pathway activation in disease, but our results show that the levels thereof indeed indicate lectin pathway activation, which of course needs to be validated in larger studies in the future.

In upcoming studies the function of C1s/C1-INH and MASP-1/C1-INH complexes should be investigated more in detail. When the complexes are formed during regulation of CP and LP activation *via* binding of C1-INH, spontaneous activation of the respective pathways and therefore consumption of C4 and C2 is limited. If the complexes also have other functions in between their release into the circulation and degradation (28) awaits further research. Besides that, C1s and MASP-1 are not the only serine proteases regulated by C1-INH. Within the complement system, C1-INH can furthermore inhibit C1r and MASP-2, also leading to the formation of covalent complexes (72, 73). Additionally, C1-INH also plays a role in other systems, such as the contact system, the fibrinolytic system as well as the coagulation system *via* binding to kallikrein, Factor XII (FXII) and Factor XI (FXI) (25). Especially the complement and the coagulation system closely interact with each other. MASP-1 for example can activate coagulation factors and thereby promote the formation of clots (74), while FXIIa/C1-INH complexes were shown to be decreased in vascular disease in SLE patients (75). If the here described C1-INH complexes might also shed light on the regulation of coagulation and thromboinflammation or if altering complex levels are indicative for a higher risk of thrombotic events still needs further research.

In summary, we have developed and validated two new sandwich immunoassays measuring C1s/C1-INH and MASP-1/C1-INH complexes in a reliable and accurate way. The new assays allow us to monitor early classical pathway activation, measured by C1s/C1-INH complexes, and early lectin pathway activation, indicated by MASP-1/C1-INH complex concentrations. For future studies, we recommend using EDTA plasma samples when measuring C1-INH complex levels in order to obtain the most reliable results. Samples should be kept on ice before the measurement and experiments should be performed within one hour after thawing to avoid additional *ex vivo* complex formation. To prevent matrix effects, samples should be diluted at least 100 times for measuring C1s/C1-INH complex levels and at least 5 times for quantification of MASP-1/C1-INH complex levels, but it is highly recommended that pilot studies are performed to determine the optimal dilution before larger sample sets are being investigated. We have set reference ranges for future applications, and our first proof-of-concept study showed that levels of both markers are increased in COVID-19, suggesting that C1-INH complex measurements might have added value for investigating and unravelling early CP and LP activation in other human diseases where complement is involved, such as SLE, HAE, Sepsis as well as other viral, bacterial and fungal infections.

## Group members of Cambridge Institute of Therapeutic Immunology and Infectious Disease-National Institute of Health Research (CITIID-NIHR) COVID BioResource Collaboration

Stephen Baker, John R. Bradley, Patrick F. Chinnery, Daniel J. Cooper, Gordon Dougan, Ian G. Goodfellow, Ravindra K. Gupta, Nathalie Kingston, Paul J. Lehner, Paul A. Lyons, Nicholas J. Matheson, Caroline Saunders, Kenneth G. C. Smith, Charlotte Summers, James Thaventhiran, M. Estee Torok, Mark R. Toshner, Michael P. Weekes, Gisele Alvio, Sharon Baker, Areti Bermperi, Karen Brookes, AshleaBucke, Jo Calder, Laura Canna, Cherry Crucusio, Isabel Cruz, Rnalie de Jesus, Katie Dempsey, Giovanni Di Stephano, Jason Domingo, Anne Elmer, Julie Harris, Sarah Hewitt, Heather Jones, Sherly Jose, Jane Kennet, Yvonne King, Jenny Kourampa, Emily Li, Caroline McMahon, Anne Meadows, Vivien Mendoza, Criona O’Brien, Charmain Ocaya, Ciro Pascuale, Marlyn Perales, Jane Price, Rebecca Rastall, Carla Ribeiro, Jane Rowlands, Valentina Ruffolo, Hugo Tordesillas, Phoebe Vargas, Bensi Vergese, Laura Watson, Jieniean Worsley, Julie-Ann Zerrudo, Laura Bergamaschi, Ariana Betancourt, Georgie Bower, Ben Bullman, Chiara Cossetti, Aloka De Sa, Benjamin J. Dunore, Maddie Epping, Stuart Fawke, Stefan Gräf, Richard Grenfell, Andrew Hinch, Josh Hodgson, Christopher Huang, Oisin Huhn, Kelvin Hunter, Isobel Jarvis, Emma Jones, Maša Josipović, Ekaterina Legchenko, Daniel Lewis, Joe Marsden, Jennifer Martin, Federica Mescia, Francesca Nice, Ciara O’Donnell, Ommar Omarjee, Marianne Perera, Linda Pointon, Nicole Pond, Nathan Richoz, Nika Romashova, Natalia Savoinykh, Rahul Sharma, Joy Shih, Mateusz Strezlecki, Rachel Sutcliffe, Tobias Tilly, Zhen Tong, Carmen Treacy, Lori Turner, Jennifer Wood, Marta Wylot, John Allison, Heather Biggs, Helen Butcher, Daniela Caputo, Debbie Clapham-Riley, Eleanor Dewhurst, Christian Fernandez, Anita Furlong, Barbara Graves, Jennifer Gray, Tasmin Ivers, Emma Le Gresley, Rachel Linger, Mary Kasanicki, Sarah Meloy, Francesca Muldoon, Nigel Ovington, Sofia Papadia, Christopher J. Penkett, Isabel Phelan, Venkatesh Ranganath, Jennifer Sambrook, Katherine Schon, Hannah Stark, Kathleen E. Stirrups, Paul Townsend, Julie von Ziegenweidt, Jennifer Webster, Ali Asaripour, Lucy Mwaura, Caroline Patterson, Gary Polwarth, Katherine Bunclark, Michael Mackay, Alice Michael, Sabrina Rossi, Mayurun Selvan, Sarah Spencer, Cissy Yong, Petra Polgarova.

## Data availability statement

The raw data supporting the conclusions of this article will be made available by the authors, without undue reservation.

## Ethics statement

The studies involving human participants were reviewed and approved by The East of England – Cambridge Central Research Ethics Committee (‘‘NIHR BioResource’’ REC ref 17/EE/0025, and ‘‘Genetic variation AND Altered Leucocyte Function in health and disease - GANDALF’’ REC ref 08/H0308/176), The Hungarian Ethical Review Agency (ETT-TUKEB; No. 8361-1/2011-EKU and IV/4403-2/2020/EKU), and The Government Office of the Capital City Budapest (31110-7/2014/EKU (481/2014)), based on the position of the Medical Research Council. The studies were conducted in accordance with the Declaration of Helsinki. The patients/participants provided their written informed consent to participate in this study.

## Author contributions

ZP and ET designed and supervised the study. LH, BB and EK performed the experiments and analyzed the data. ZP, GS, PL and LB were involved in recruitment and sampling of healthy controls and patients. ET and LH wrote the first draft of the manuscript. ZP, EK, BB, RB, WB, PL, LC, and RW critically reviewed the manuscript. All authors contributed to the article and approved the submitted version.

## Funding

The research was financed by the Higher Education Institutional Excellence Programme of the Ministry of Human Capacities in Hungary, within the framework of the molecular biology thematic program of the Semmelweis University, “MOLORKIV”(TKP2021-EGA-24) to ZP. ZP, RW and LH are supported by funds of the EU MSCA project CORVOS 860044. The research was further funded by the Austrian Science Fund (FWF), HOROS W-1253. In addition, the research project (project number: 2020-1.1.6-JÖVŐ-2021-00013 to ZP) was further funded by the Ministry of Culture and Innovation with support from the National Research Development and Innovation Fund under the Investment into Future call (2020-1.1.6-JÖVŐ) programme.

## Acknowledgments

We acknowledge the technical assistance of Márta Kókai, Éva Zsuzsanna Szendrei, Lászlóné Kertész, Edina Szabó and Beáta Takács, and the help of Veronika Makó during development of monoclonal antibodies with many thanks. We further thank Dorottya Csuka for the help with creating the figure using BioRender. Parts of the work were presented at the 28^th^ International Complement Virtual Workshop (ICW2021) and the 18^th^ European Meeting on Complement in Human Disease (EMCHD2022).

## Conflict of interest

ET, BB, RB and WB are employees of Hycult Biotech.

The remaining authors declare that the research was conducted in the absence of any commercial or financial relationships that could be construed as a potential conflict of interest.

## Publisher’s note

All claims expressed in this article are solely those of the authors and do not necessarily represent those of their affiliated organizations, or those of the publisher, the editors and the reviewers. Any product that may be evaluated in this article, or claim that may be made by its manufacturer, is not guaranteed or endorsed by the publisher.
