# Corrigendum: Distinction of early complement classical and lectin pathway activation *via* quantification of C1s/C1-INH and MASP-1/C1-INH complexes using novel ELISAs

**DOI:** 10.3389/fimmu.2025.1561850

**Published:** 2025-02-12

**Authors:** Lisa Hurler, Erik J. M. Toonen, Erika Kajdácsi, Bregje van Bree, Ricardo J. M. G. E. Brandwijk, Wieke de Bruin, Paul A. Lyons, Laura Bergamaschi, Cambridge Institute of Therapeutic Immunology Infectious Disease-National Institute of Health Research (CITIID-NIHR) COVID BioResource Collaboration, György Sinkovits, László Cervenak, Reinhard Würzner, Zoltán Prohászka

**Affiliations:** ^1^ Department of Internal Medicine and Haematology, Semmelweis University, Budapest, Hungary; ^2^ Research and Development Department, Hycult Biotech, Uden, Netherlands; ^3^ Cambridge Institute of Therapeutic Immunology and Infectious Disease, Jeffrey Cheah Biomedical Centre, University of Cambridge, Cambridge, United Kingdom; ^4^ Department of Medicine, University of Cambridge, Addenbrooke’s Hospital, Cambridge, United Kingdom; ^5^ Institute of Hygiene and Medical Microbiology, Medical University of Innsbruck, Innsbruck, Austria; ^6^ Research Group for Immunology and Haematology, Semmelweis University – Eötvös Loránd Research Network (Office for Supported Research Groups), Budapest, Hungary

**Keywords:** early complement activation, classical pathway activation, lectin pathway activation, C1-INH complexes, assay development and validation, C1s/C1-INH complex, MASP-1/C1-INH complex

In the published article, there was an error in the **Funding** statement. One of the funding sources was missing from the original published statement. The correct Funding statement appears below. “The research was financed by the Higher Education Institutional Excellence Programme of the Ministry of Human Capacities in Hungary, within the framework of the molecular biology thematic program of the Semmelweis University, “MOLORKIV”(TKP2021-EGA-24) to ZP. ZP, RW and LH are supported by funds of the EU MSCA project CORVOS 860044. The research was further funded by the Austrian Science Fund (FWF), HOROS W-1253. In addition, the research project (project number: 2020-1.1.6-JÖVŐ-2021-00013 to ZP) was further funded by the Ministry of Culture and Innovation with support from the National Research Development and Innovation Fund under the Investment into Future call (2020-1.1.6-JÖVŐ) programme.”

The authors apologize for this error and state that this does not change the scientific conclusions of the article in any way. The original article has been updated.

